# Culture influences conscious appraisal of, but not automatic aversion to, acoustically rough musical intervals

**DOI:** 10.1371/journal.pone.0294645

**Published:** 2023-12-05

**Authors:** James Armitage, Imre Lahdelma, Tuomas Eerola, Rytis Ambrazevičius

**Affiliations:** 1 Music Department, Durham University, Durham, United Kingdom; 2 Kaunas University of Technology, Kaunas, Lithuania; Tohoku University: Tohoku Daigaku, JAPAN

## Abstract

There is debate whether the foundations of consonance and dissonance are rooted in culture or in psychoacoustics. In order to disentangle the contribution of culture and psychoacoustics, we considered automatic responses to the perfect fifth and the major second (flattened by 25 cents) intervals alongside conscious evaluations of the same intervals across two cultures and two levels of musical expertise. Four groups of participants completed the tasks: expert performers of Lithuanian Sutartinės, English speaking musicians in Western diatonic genres, Lithuanian non-musicians and English-speaking non-musicians. Sutartinės singers were chosen as this style of singing is an example of ‘beat diaphony’ where intervals of parts form predominantly rough sonorities and audible beats. There was no difference in automatic responses to intervals, suggesting that an aversion to acoustically rough intervals is not governed by cultural familiarity but may have a physical basis in how the human auditory system works. However, conscious evaluations resulted in group differences with Sutartinės singers rating both the flattened major as more positive than did other groups. The results are discussed in the context of recent developments in consonance and dissonance research.

## Introduction

### Psychoacoustic vs cultural explanations of consonance and dissonance

The foundations of musical consonance and dissonance have received scholarly interest since the days of Pythagoras in ancient Greece [1], yet there is still no consensus as to whether they are a biological universal or culture specific. The notion of consonance and dissonance is fundamental to (Western) music theory, but there is still no fully accepted consensus of exactly what constitutes this categorical perception of musical sounds. Indeed, much of the empirical research into consonance and dissonance (hereafter referred to as C/D an implying exclusively simultaneous, not successive sounds) is confounded by related concepts such as pleasantness, valence and preference (for an over view, see e.g., [2]). Broadly speaking, explanations of C/D are typically rooted in some combination of psychoacoustics and culture. Psychoacoustic accounts of C/D explain it in terms of acoustic measures such as *roughness*, *harmonicity*, and *sharpness* [3, 4], which are calculated based on the properties of the sounds themselves, and their interaction with the human auditory system. Perceptual roughness results when a complex tone displays a rapid amplitude modulation. This is a result of partials of a complex tone that are too close together to be resolved fully by the basilar membrane [5], but not sufficiently close to be perceived as a unison. Harmonicity in turn indicates how closely a sonority’s spectrum corresponds to a harmonic series [6]. Finally, sharpness denotes the energy at high frequencies which has also been identified as a predictor of C/D [7, 8].

Sound combinations which are high in roughness are typically, though not universally, perceived as unpleasant on an aesthetic level. While Western listeners commonly perceive roughness as disagreeable [9–11] it is harnessed for aesthetic ends (in moderate amounts) in the vocal practice of *beat diaphony* (also known in ethnomusicological literature by the German term *Schwebungsdiaphonie*) which refers to a two-part singing performance style where intervals of parts form predominantly rough sonorities and audible beats [12, 13]. It is present in diverse parts of the world, for example, the Baltic (Lithuanian Sutartinės) and the Balkan (e.g., Bosnian Ganga) regions of Europe [12, 14] as well as in Papua New Guinea [15], Nepal, Afghanistan, Ethiopia [16], and the Indonesian islands [17].

In addition to purely psychoacoustic accounts, there is empirical evidence to suggest that C/D is also influenced by culture; recent research has also pointed out that psychoacoustics and culture may overlap as in the case of harmonicity and cultural familiarity (see [18, 19]). In terms of cross-cultural experiments, Maher [20] found that North Indian listeners rated dissonant intervals as markedly less tense than Western (Canadian) listeners, in line with the notion that musical intervals classed as highly dissonant in Western music are used more freely in North Indian classical music [21]. McDermott et al. [22] in turn conducted fieldwork with the Bolivian Tsimané people, who are to a large extent insulated from Western cultural influences. The study concluded that the Tsimané are indifferent to C/D, although highly dissonant chords were omitted from the stimuli which somewhat hinders the results’ generalisability; moreover, on closer inspection it is evident that the Tsimané did in fact have an aversion to the minor and major second intervals when presented diotically (simultaneous presentation to each ear). Despite this, McDermott and colleagues argued that on the basis of their results the foundations of C/D are purely cultural rather than psychoacoustic. More recently, Lahdelma et al. [9] conducted fieldwork in remote Northwest Pakistan on how the minimally Westernised members of the Khalash and Kho tribes perceive harmony. Applying a wider range of C/D than McDermott et al. and using a direct selection task and pictorial representations to circumvent some of the semantic confounds that have been posing challenges for cross-cultural research, Lahdelma et al. [9] found that there was an aversion to the highly rough chromatic cluster chord across both Western and the Khalash/Kho listeners, but that the preference for the consonance of the major triad was present only in Western listeners.

Taken together, there is an apparent conflict between the psychoacoustic predisposition for an automatic aversion to acoustically rough intervals as recently demonstrated by Armitage et al. [23] with the preference for dissonant harmonic combinations that occur in the vocal practice of *beat diaphony* in many non-Western musical traditions [12, 14]. Armitage et al. argue that automatic responses to dissonant intervals are driven by interactions of fundamental frequencies in the basilar membrane—i.e., when the fundamental frequencies of a complex tone lie within the same critical bandwidth and the basilar membrane is unable to resolve them, resulting in an unpleasant sensation. The width of the critical band varies with the centre frequency, but perceptual roughness occurs maximally when the frequencies are around 25% of a critical bandwidth apart [24]. The critical bandwith around middle C is roughly 100 Hz or a perfect fifth [25]; maximal perceptual roughness occurs at roughly 25 Hz or just less than a major second. The critical band has long held a central place in C/D theories, beginning with Helmholtz [26], and underpins modern models of roughness. As demonstrated by Armitage et al., intervals high in acoustic roughness, such as the minor and major seconds, are processed automatically as negative in the case of Western listeners. Other intervals which have conventionally negative connotations in Western music, e.g. the ‘sad’ minor third, or the ‘diabolic’ tritone, are understood as negative as a result of culture. Such intervals are typically rated as unpleasant or negative in conscious self-report evaluations [27, 28] but not in the case of paradigms that probe automatic, unconscious responses to acoustic features [23]. Interestingly, Maher [20] questions the physiologically-based critical band theory’s validity to account for the aversion to dissonant intervals based on the very finding that North Indian listeners perceived highly dissonant intervals as remarkably neutral compared to Canadians; indeed Maher (p. 271) proposes that “it does not seem likely that basilar membrane characteristics would be culture-specific”.

It is crucial to note however that all of the previous cross-cultural experimental procedures into the question of C/D have used exclusively self-report measures—participants rated their impressions of the stimuli on a scale [20, 22], or through preference choices and pictorial assessment [9]. An important—and yet unresolved—question is whether these responses mirror or diverge from responses that happen more automatically without any opportunity for conscious appraisal of the stimuli. Automatic or implicit responses to consonant and dissonant stimuli have been investigated using reaction time (RT) and event-related potential (ERP) methods. Over the last 20 years, a number of studies have used affective priming techniques as a measure of attitudes to musical chords; priming studies using chord targets and word primes have found a strong consensus that hearing a dissonant three- or four-note chord facilitates processing of negative words, whereas hearing a consonant chord facilitates processing of positive words [29–31]. However, in the case of intervals the facilitation effects only seem to be present when the intervals used contrast maximally in roughness [23]. In particular, this effect has been observed when intervals that lie within the critical bandwidth (i.e., the minor and major seconds) have been used as the negative prime intervals, but not when other highly dissonant wide intervals such as the major seventh have been used.

Importantly, the questions of whether these automatic responses are subject to variation across cultures, and of whether and to what extent these variations mirror variations in self-report measures have yet to be addressed. Or to reiterate the sceptical view of Maher [20] on the critical band theory, it is unclear whether mere exposure through cultural familiarity may override the physical and possibly universal response to the negative valence of dissonant intervals that lie within the critical bandwidth (see Armitage et al. [23]). One way of answering these questions is to utilise the priming paradigm with participants who are regularly exposed to what are considered dissonant intervals in Western music (and which are within the critical bandwidth), such as the minor and major seconds. Lithuanian Sutartinė singers is one such group that fulfils this criterion.

### Sutartinės

**Fig 1:**
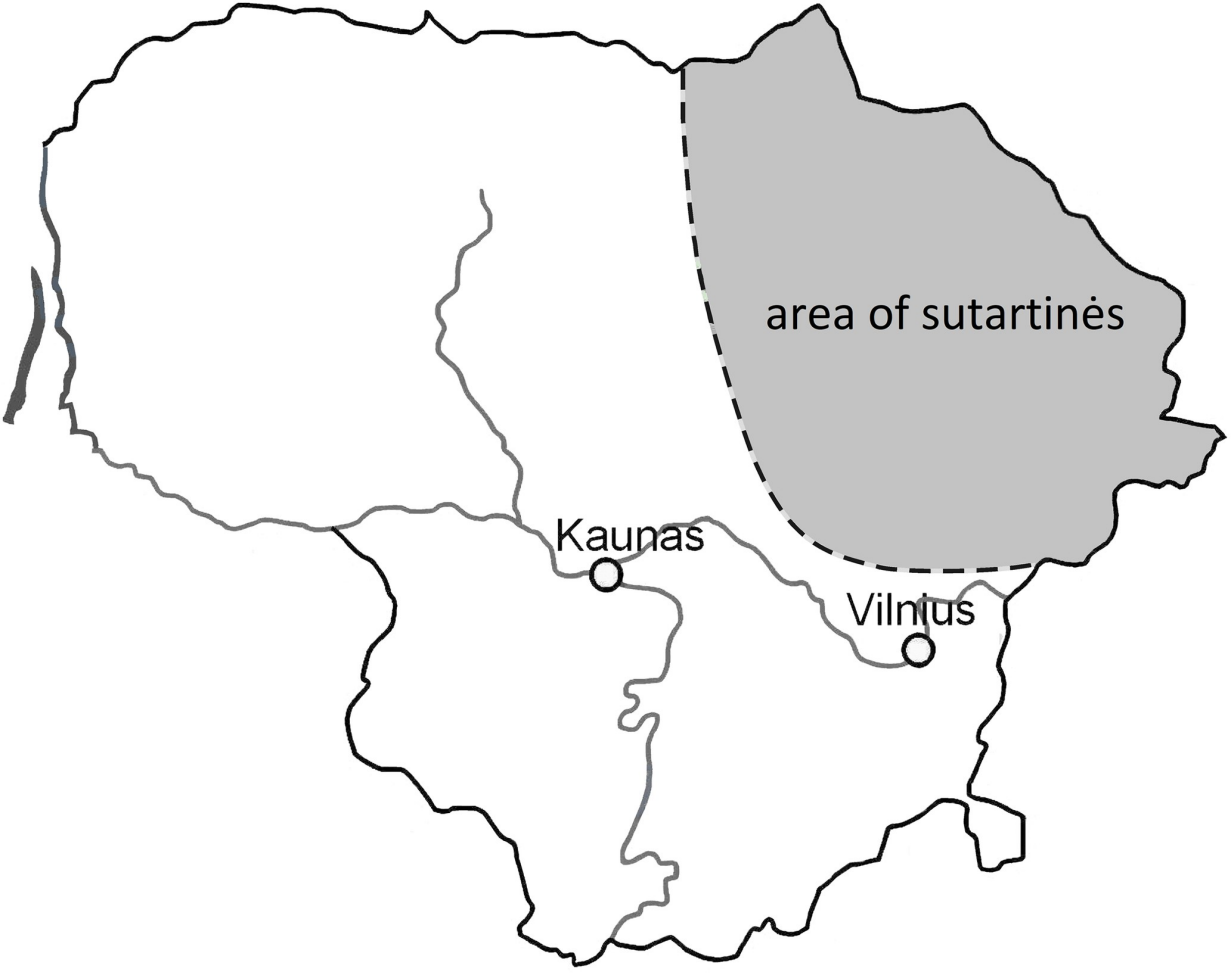
Map highlighting Sutartinės area within Lithuania. Republished from [35] under a CC BY license, with permission from Universität Bern, original copyright 2022.

**Fig 2:**
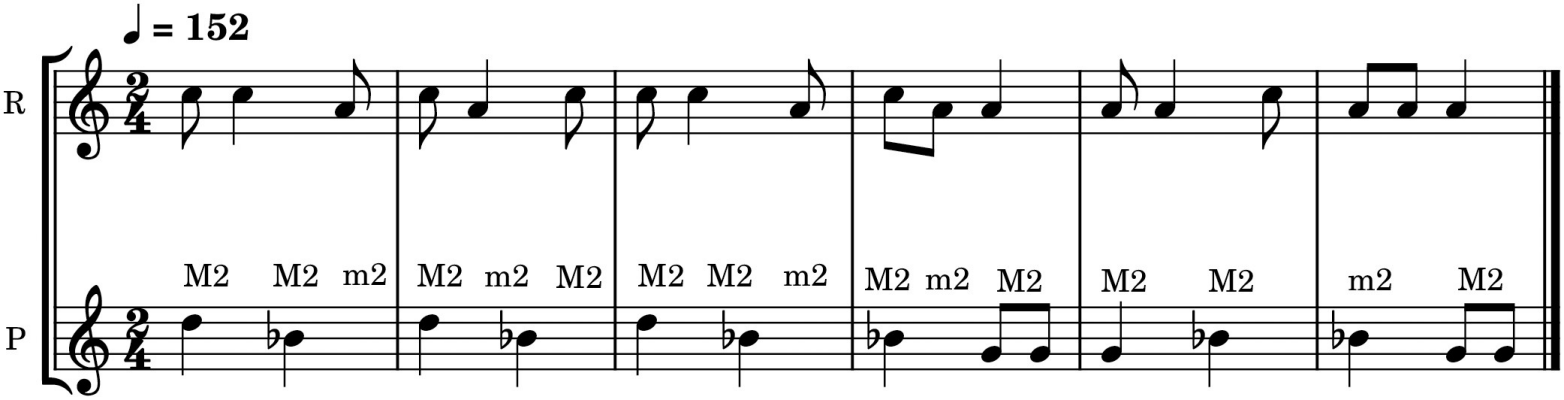
Example of Sutartinė singing: An excerpt from “Myna, Myna, mynagauc̆io lylio” (adapted from Ambrazevičius et al. (2015) [13], p. 221.); the major and minor seconds in Western diatonic notation approximate the ‘squeezed’ major seconds in the Sutartinės vocal style.

The Lithuanian Sutartinė style is an unaccompanied vocal style, sung by female singers, originating in Northeastern Lithuania (see Fig 1). The name derives from the verb *sutarti* which means ‘to agree’, ‘to be in concord’ [12]. It is characterised by a narrow melodic range, the prevalence of dissonances (particularly ‘squeezed’ major seconds, i.e. somewhat flatter than a (12-TET) major second, see e.g. Ambrazevičius [32]) and heterophonic textures in three parts (although two- and four-part Sutartinės are also common). Fig 2. provides an example of a typical vocal texture in this style. The major and minor seconds are utilised for expressive purposes; notably, the rough ‘clashes’ of the seconds are positively connoted and are appreciated for their ‘bell-like’ sound [12] and this connotation is also known in the vocal traditions of Bulgaria, Serbia, Bosnia-Herzegovina, Macedonia, South Albania, Romania, and Northern Greece [16]. This analogy presumably arises from similar psychoacoustic qualities including close partials, beats, attacks, and frequency range (see [12, 33]). Although vocal Sutartinės are more common, there also exists and instrumental Sutartinės tradition [34] that employs traditional Lithuanian woodwind instruments.

Sutartinės reached near-extinction in the mid 20th century [32, 36], but have since gained popularity amongst folk music groups as a part of the broader revival in interest in the folklore and music of the Baltic countries [37]. Ambrazevičius & Wiśniewska [32] probed the tonal hierarchies of a corpus of Sutartinės and reported that Sutartinės singers exhibited different cognitive representations of the tonal hierarchies, for instance attributing greater salience to the two central notes in the Sutartinės scale—akin to a “double tonic” effect—compared to controls and they also attributed much lower salience to the more distant notes form the tonic compared to controls. This is consistent with Krumhansl and Castellano’s [38], schema-based account of music perception. In particular, they present the idea that there is a relationship between perception of musical events and a listener’s knowledge of the underlying structures of the musical style. Specifically with regard to Sutartinės, Ambrazevičius & Wiśniewska’s [32] findings suggest that listeners with expertise in Sutartinės have a more distinct cognitive representation of the tonal hierarchies of Sutartinės than listeners without such expertise.

### Affective priming

In affective priming tasks, participants are exposed to two stimuli in quick succession (the *prime*, here an interval, and the *target*, in this case a word). Participants classify the second (target) stimulus as positive or negative. Responses to affective priming tasks are generally accepted to be providing an implicit measure of attitudes to a stimulus (see e.g., [39, 40]). In particular, affective priming is well established as a measure of automatic responses to C/D [29–31]. There is consensus in the music priming literature that targets are classified more quickly and accurately when the chord and the word are of the same valence (i.e. dissonant-negative or consonant-positive) compared to when the stimulus and the word are of opposing valence.

### The present study

The present study aims to investigate how listeners’ expertise in a musical tradition with a high prevalence of acoustically rough intervals (i.e. the minor and major second in Lithuanian Sutartinės) influences their responses to these intervals compared to non-musicians and musicians trained in Western diatonic genres. In particular, we test whether there is a differential effect on automatic responses (which we predict are governed by psychoacoustic factors) and conscious evaluative responses (which we predict are governed by familiarity.) More specifically, we predict that automatic responses to an affective priming task are independent of musical background, whereas appraisal responses are be influenced by expertise in Sutartinės, i.e. by exposure to a particular musical culture in which ‘squeezed’ major second intervals are more prevalent. The influence of a musical culture rich in acoustic roughness on C/D perception is compared with the influence of expertise in Western tonal music, where acoustically rough intervals are used less frequently. We expect Sutartineės singers to rate the major second as more positive than do Western musicians, who we in turn expect to rate the major second as more positive than non-musicians due to higher familiarity. In the affective priming task, we expect that targets would be classified more quickly and more accurately when they are preceded by a congruent prime. However, we do not predict any group differences in this effect.

## Materials and methods

### Participants

Brysbaert and Stevens [41] suggest that, for a mixed design with multiple items in each condition, 1600 readings per condition (i.e., 25 participants per group) are necessary to achieve a power of 0.8. To mitigate against attrition, we targeted a sample size of thirty per group. Following deletions (see data pre-treatment below), the Sutartinės group consisted of 24 members of Lithuanian Sutartinės singing groups (all female; mean age = 42.4, SD = 13.6; slightly below target sample size). The control group consisted 26 female native speakers of Lithuanian recruited via Prolific.co, an online crowd-sourcing platform designed especially for research purposes (mean age = 29.09, SD = 6.09). The Western musician and non-musician groups were also recruited via Prolific. The musician group were pre-filtered as having at least 5 years’ experience in singing/playing an instrument. 28 musicians (all female, mean age = 46.5 years, SD = 15.2) and 31 non-musicians (all female, mean age = 37.2 years, SD = 11.5) completed the study. Participants received £3.25 (€3.69) for participating in the study. The study received ethical approval from the Department of Music ethics committee, University of Durham (MUS-2021-02-04T10_57_12-ghth52). Informed consent was provided via an online checkbox.

### Materials

**Fig 3:**
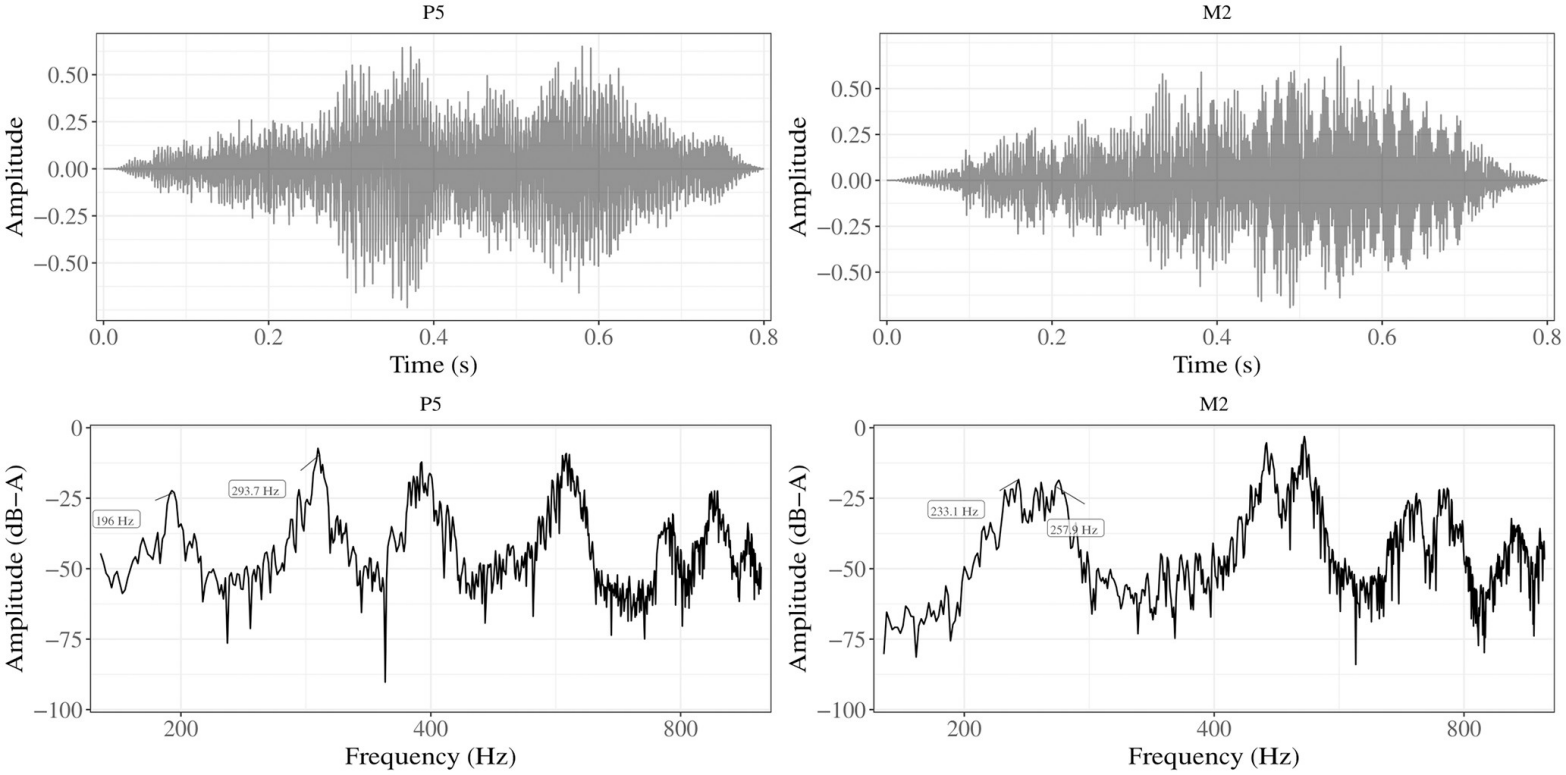
Waveforms and spectra for P5 and M2.

To test automatic versus conscious appraisal responses to the intervals, we used two intervals: a perfect fifth (*G*_3_ − *D*_4_) (P5) and a flat major second (*Bb*_3_ − *C*_4_) (M2) tuned to 175 cents, consistent with the Sutartinės style which harnesses intervals slightly narrower than the equally-tempered major second, with a wide range of variations [12]. The M2-P5 combination has previously proven effective at generating affective priming effects [23]. The intervals were generated with *Ableton Live 9* (a music sequencer software), using the *Venus Symphonic Women’s Choir* sample-based plug-in as the sound font. The stimuli were normalised by setting them to the same peak sone level. The duration of the stimuli were 800 ms with a 50 ms fade-out. The amplitudes and spectra for the two intervals are shown in Fig 3.

Target words in English and Lithuanian are listed in Table 1. The words were translated from English to Lithuanian by the fourth author; back translation to English by an independent native speaker of Lithuanian agreed with the original words. As each prime interval was paired with each target word, there were 32 possible prime-target combinations. Each combination could be either congruent (e.g. P5—“lively”, or M2—“morgue”) or incongruent (e.g. P5—“dismal”, M2—“excite”).

**Table 1:** Target words in English and Lithuanian.

English	Lithuanian
climax	kulminacija
lively	gyvas
gentle	švelnus
rest	ilsėtis
comfy	patogus
admire	žavėtis
payday	atlyginimo diena
relax	atsipalaiduoti
rabid	pasiutęs
hijack	pagrobti
coma	koma
arrest	areštuoti
flaccid	bevalis
morgue	morgas
coward	bailys
dismal	niūrus

The priming experiments and the rating task were implemented online in PsyToolkit [42]. RT data collected via PsyToolkit and Prolific have previously been shown to be comparable to lab-based data collection [43].

### Procedure

Participants were told that they were completing an experiment on the relationship between sounds and reading. Initially participants provided demographic information and details of their musical background and expertise. Next, in order to orient them to the musical style [44], participants heard a short extract from the Sutartinės *Atvažiuok, močiute* and *O kas pražydo* which lasted a total of 2 minutes 45 seconds.

For the priming task, participants were presented with a series of target words which were to be categorised as either positive or negative. The task was restricted so that participants could only use laptop or desktop computers with a physical keyboard, i.e. it was not possible to complete the task via mobile phone or tablet. Participants were instructed to press the ‘z’ key if the word had negative connotations or the ‘m’ key if the word had positive connotations. Each word was preceded by a brief sounding of an interval. The timings for each item of the priming task was as follows. Initially, a fixation cross was presented on-screen. After 450 ms, the prime intervals sounded for 800 ms. 200 ms into the auditory presentation of the prime interval, the fixation cross was replaced with the target word, which remained onscreen for 2000 ms or until a key press.

The priming task consisted of a ten-item practice block, followed by two 64-item (i.e. two prime chords x 16 target words; each prime-target combination occurred twice) experimental block. During the practice block, participants received feedback after each item to indicate whether or not their answer was correct; no feedback was given during the experimental block. Items were presented in a random order.

For the rating task, participants rated the M2 and P5 intervals on a 7-point likert scale where 1 represented most negative and 7 represented most positive. Following the experiment, participants were debriefed as to the purpose of the study.

## Results

### Data pre-treatment and statistical analysis

Prior to analysis, five participants were removed from the data set in line with the exclusion criteria above. Incorrect responses to individual items were deleted from the data set, as were timeouts (i.e. instances where participants fail to classify the target word within 2000 ms). Each participant’s data was fitted to a Gamma distribution: responses to individual items that were faster than 250 ms or slower than the 95th percentile of the Gamma distribution were deleted from the data set.

Reaction time data were fitted to a generalised mixed model [45], using the R library lme4 [46] with the fixed effects of Group and Congruence (congruent vs incongruent), with participant as random effect. The model was then subjected to a 4 (Group) × 2 (Congruence: congruent vs incongruent) type III ANOVA. Standardised effect sizes are not reported for GLMM. All statistical analyses were carried out in R [47] at *α* = .05. Where there are multiple comparisons, Bonferroni corrections have been used throughout.

### Reaction time analysis

Mean reaction times (standard deviations) and accuracy rates (%) are given in Table 2.

**Table 2:** Mean (SD) reaction times and accuracy rates per condition.

	Sutartinės Singers	Lithuanian Non-musicians	Western Musicians	Western Non-musicians
Congruent	691 ms (147) 90.5% (5.14)	654 ms (175) 87.8% (5.61)	615 ms (115) 88.4% (3.82)	638 ms (150.0) 87.0% (6.60)
Incongruent	706 ms (143) 88.2% (6.85)	662 ms (176) 88.2% (5.85)	621 ms (112) 85.6% (4.69)	648 ms(151) 85.8% (5.33)

**Fig 4:**
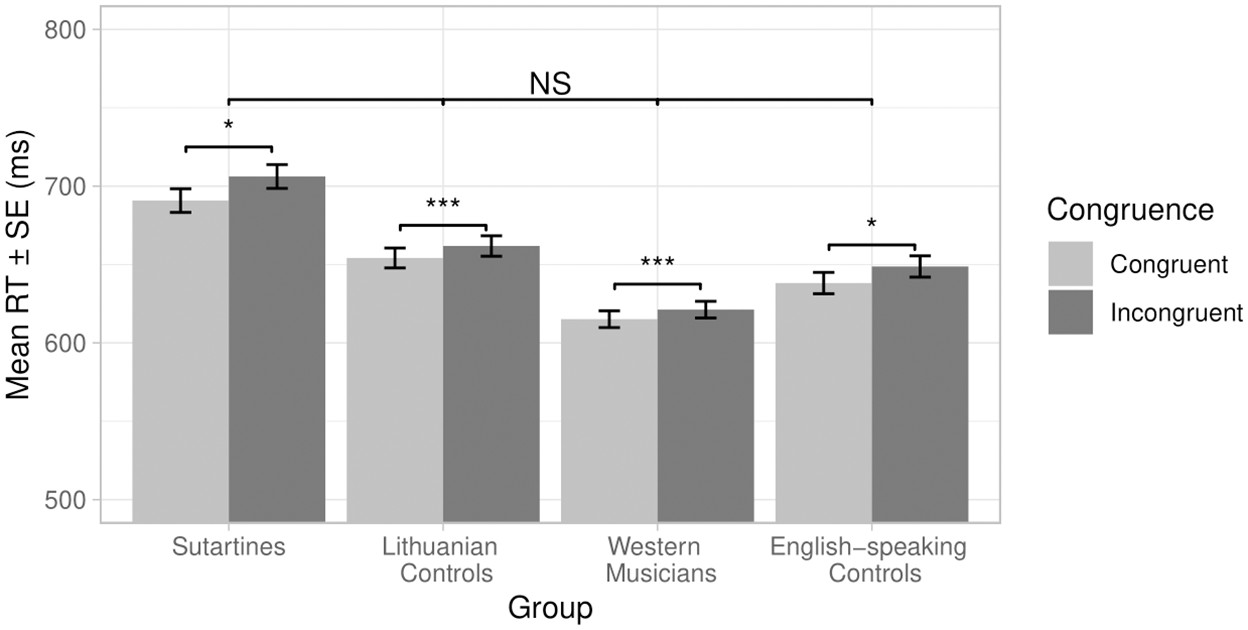
Mean and SE RT by congruency condition. Between groups differences were non-significant; for within groups differences, * denotes *p* < .05 *** denotes *p* < .001.

The ANOVA revealed a significant main effect of congruency, *F*(1, ∞) = 32.78, *p* < .001. Planned contrasts showed that RTs in the Congruent condition were significantly faster than those in the Incongruent condition, *z* = 5.73, *p* < .0001, 95%*CI* = [0.25, ∞]. The congruency effect is represented graphically in Fig 4. The main effect of Group proved non-significant, *F*(3, ∞) = 1.43, *p* = .23, as did the interaction of Congruence and Group, *F*(3, ∞) = 1.15, *p* = .33. The GLMM is summarised in Table 3.

**Table 3:** Summary of GLMM for RT.

Factor	*β*	95% CI	SE	t-value	p-value
Intercept (The intercept corresponds to Lithuanian Non-musicians in the congruent condition)	6.74	[6.44, 7.03]	0.15	44.99	<.001
Incongruence	-0.04	[-0.06,-0.03]	0.008	-5.73	<.001
Western Non-musicians	0.00	[-0.51, 0.52]	0.26	1.582	0.1136
Sutartineės Group	-0.08	[-0.58, 0.43]	0.149729	0.26	0.8188
Western Musicians	0.47	[-0.05, 0.98]	0.26	1.78	0.08
Incongruence × Western Non-musicians	0.01	[-0.01, 0.04]	0.01	-0.416	0.36
Incongruence × Sutartinės	-0.009	[-0.03, 0.02]	0.01	-0.229	0.49
Congruence × Western Musicians	-0.02	[-0.05, 0.01]	0.01	1.307	0.19

### Accuracy rate analysis

Next, accuracy rates were subjected to a 4 (Group) × 2 type III ANOVA. The main effect of Congruence proved significant, *F*(1, 105) = 10.26, *p* = .002. Planned contrasts confirmed that ARs in congruent conditions were significantly higher than in incongruent conditions, *t*(108) = 3.16*p* = .001, 95%*CI* = [0.82, ∞, *d* = 0.31 The main effect of Group proved non-significant *F*(3, 105) = 0.82, *p* = .49. The interaction of Congruence and Group was also non-significant, *F*(3, 105) = 1.92, *p* = .13.

### Valence ratings

Mean (SD) valence ratings for the stimuli are given in Table 4.

**Table 4:** Mean (SD) Valence ratings for audio stimuli.

Interval	Sutartinės Singers	Lithuanian Non-musicians	Western Musicians	Western Non-musicians	Overall
M2	3.54 (1.74)	2.11 (1.03)	2.87 (1.38)	3.36 (1.50)	2.97 (1.51)
P5	5.67 (1.20)	4.73 (1.76)	5.14 (1.18)	5.19 (1.33)	5.15 (1.41)

Finally, we considered the valence ratings for M2 and P5 from the Lithuanian and the English-speaking groups. The data were subject to a MANOVA with Pillai’s trace, with the dependent variables M2 and P5 valence ratings and the independent variable of group.

We saw a significant main effect of Group, *V*(6, 212) = 2.9518, *p* = .008. Subsequent univariate ANOVAs yielded a significant effect of group on M2 ratings, *F*(3, 106) = 5.30, *p* = .002. We used a planned contrasts approach to test our hypothesis that familiarity with M2 would lead to higher valence ratings, i.e., Sutartinės singers would rate M2 higher than Western musicians who would, in turn rate M2 as more positive than non-musicians. Planned contrasts were significant, *t*(106) = 2.089, *p* = .02, 95%*CI* = [0.37, ∞].

## Discussion

Automatic responses to musical intervals were found to follow a similar pattern, irrespective of musical culture or level of musical expertise. Congruency effects were found to be present in all four groups of participants, i.e., reaction times were shorter when the musical interval and the target word both had the same valence compared to when the musical interval and the target word had opposite valences and this finding is in line with recent research investigating the perception of C/D in intervals with an affective priming paradigm targeting Western listeners [23, 30]. In particular, the Sutartinės singers did not differ in their responses from the other groups. This suggests that exposure to a musical culture where there is a high prevalence of parallel seconds does not influence automatic responses to those intervals. Previous research has argued that roughness is the governing factor in automatic responses to intervals and more complex chords [23, 30, 31]. In these instances, the negative valence allocated to ‘rough’ intervals such as seconds is thought to be a consequence of the inability of the basilar membrane to resolve the fundamentals waves that are close in frequency—i.e., which lie within the same critical band—and is therefore not susceptible to modulation by musical experience.

In contrast to the automatic responses to the intervals, valence ratings did reveal group differences. As predicted, Sutartinės singers rated the flattened major second as more positive than did their Western musician counterparts or participants in the Lithuanian and Western non-musician groups. Although this presents an apparent contradiction with the result of the reaction time task, it seems likely that the valence ratings are based on a conscious mechanism that depends on acquired knowledge of particular musical sound-worlds, which allows for a more positive interpretation of the M2 interval, whereas this knowledge based understanding of the interval is not activated during the priming task. In such tasks the response is formulated at an earlier stage of processing, i.e., the evaluation as negative is in response to activity in the basilar membrane. This finding is in line with both neurological studies [48, 49] as well as affective priming studies [23, 30, 31] that have not found a difference in automatic reactions to consonant vs. dissonant (rough) pitch combinations among Western listeners, even if the conscious appraisal of such pitch combinations does differ according to musical expertise.

It is notable that Sutartinės singers rated P5 as more positive than the other groups did. It seems plausible that this is a consequence of the Sutartinės singers’ higher familiarity with the idiomatic female choir timbre, which they may associate with the Sutartinės singing style and hence find it more positive in general than listeners without familiarity with this style. Previous research has established that more familiar timbres may indeed be perceived as more positive in terms of valence and preferred more than unfamiliar ones (see Lahdelma & Eerola [2] for differences between the perception of sine-wave and piano timbres). With a timbre less intrinsically associated with the particular musical experience of one of the groups (e.g., piano), there would perhaps not be such a difference between the musically sophisticated listeners across the two distinct cultural groups. Despite a more positive conscious evaluation of both the M2 and P5 intervals among the Sutartinės singers it is likely that this does not affect the automatic responses that capitalise on the contrast between the two intervals and the positive and negative words, as indicated by the lack of statistically significant differences between all four participant groups in this regard.

Although recent fieldwork across non-Western and Western populations has identified high amounts of roughness as a possible source for universal aversion in simultaneous pitch-combinations [9], it has also been proposed that musical sounds are not rough enough to cause aversion that is determined by the properties of the human auditory system [22]. Indeed, the validity of the critical bandwidth theory has been questioned [20] on the basis of findings that many non-Western musical cultures promote roughness for aesthetic ends in musical expression. Here we have demonstrated that these apparently contradictory findings may be reconciled if we separate the layers of acquired aesthetic, self-reported responses and automatic affective responses that are likely to be biologically determined due to constraints of the human auditory system.

Notably, the automatic aversion to the M2 interval found here is in line with McDermott et al.’s finding that the un-Westernised Tsimané people also had an aversion to major and minor seconds (i.e., the two intervals whose separate fundamentals fall within the same critical band) when presented diotically (simultaneous presentation to each ear) [22]. The Tsimané provide a particularly compelling case as in terms of exposure to simultaneous pitch combinations they can be considered as a *tabula rasa*—vertical harmony does not feature in the Tsimané musical culture. Hence, their responses are not likely to be the consequence of familiarity with either Western tonal harmony, or conversely, the rough harmonies prevalent in beat diaphony musical styles. Of additional interest here is the research of McPherson and colleagues [50] conducted also on the Tsimané demonstrating that perceived fusion (the tendency for simultaneous sounds to blend perceptually or to be perceived as one sound) among the Tsimané was greater for the intervals of the octave, the fifth, and the fourth than for the dissonant intervals closest in size, just like in the case of Western listeners. Strikingly, fusion did not predict preferences in Tsimané participants, who did not indicate a preference for these fused (consonant) intervals. This reiterates the previous point that while the distinction between consonance/dissonance may have a biological basis in how the human auditory system works, this distinction does not entail a universal aesthetic response on a conscious level.

The present study addressed C/D in the context of Sutartinės singers and musicians in Western tonal traditions alongside non-musician controls from Lithuania and English-speaking countries. However, its scope is limited to intervals rather than more complex chords. It should also be noted that harmony perception is not usually limited to isolated intervals (or chords), i.e., harmony should be considered horizontally as well as vertically. As Persichetti (p. 189) points out these two planes interact dynamically in actual music: “when melodies sound together chords are formed, and when chords follow each other melodic motion is involved…even the most isolated chord is full of melodic potential”. [51] It is clear that C/D is affected by context as well as musical style: for example what is restless and dissonant in common-practice tonality may be considered relaxed and consonant in jazz, as in the case of dissonant chords giving a sense of finality [52]. Future research should also expand the scope of cultures considered in perception of C/D. In particular, practitioners of other roughness-rich musical traditions such as Ganga (Bosnia), Gamelan (Indonesia), Mijwiz (the Middle East) would provide fertile ground for exploring the relationship between C/D, biological factors, and culture. In addition, other musical features, such as timbre, whose perception could be influenced by both biological and cultural factors should be studied using a similar combination of experimental and self-report methods.
